# Supplementary material for: Carrion Crows and Azure-Winged Magpies Show No Prosocial Tendencies When Tested in a Token Transfer Paradigm
Source: Animals (Basel). 2021 May 24;11(6):1526. doi: 10.3390/ani11061526 (PMC8225188; doi:10.3390/ani11061526)
Supplement: Supplementary file 1 [file animals-11-01526-s001.zip › Horn et al. PROEX Supplementary Information File 1_NEW.pdf]

**Supplementary Information File 1**

# Carrion crows and azure-winged magpies show no prosocial tendencies when tested in a token transfer paradigm

Lisa Horn, Jeroen S. Zewald, Thomas Bugnyar, Jorg J. M. Massen

## Supplementary Results - Recipient Behavior

Table S1 shows the behavior scores for each individual carrion crow recipient. Note that these scores do not necessarily sum up to the overall scores of "one or more recipients close to the given location" presented in the main manuscript, because in some cases several recipients were close to the respective locations simultaneously.

**Table S1.** Duration that each carrion crow recipient spent close to the wire mesh partitioning a) in the social control and b) in the test, as well as c) close to the exchange table in the test. All scores are averaged across the two sessions of each condition. Female birds are highlighted in light purple color.

| **a)** | | **RECIPIENT - Close to the Wire Mesh Partitioning in SOCIAL CONTROL** | | | | | | | | |
|---|---|---|---|---|---|---|---|---|---|---|
| | | Juno | Signore | Soukie | Daisy | Paula | Peppi | Saul | Walter | Willi |
| **DONOR** | Caruso | 2.5 | 0.0 | 0.0 | 112.8 | 62.8 | 0.7 | 0.0 | - | - |
| | Daisy | - | 0.0 | 0.0 | - | 2.0 | 0.0 | 35.6 | - | - |
| | Paula | - | 120.5 | 0.0 | 0.0 | - | 53.5 | 3.9 | - | - |
| | Peppi | - | 0.0 | 0.0 | 0.0 | 0.0 | - | 0.0 | - | - |
| | Saul | - | 0.0 | 5.4 | 115.1 | 0.0 | 0.0 | - | - | - |
| | Walter | - | 0.0 | 0.0 | 18.6 | 8.7 | 7.9 | 0.0 | - | 21.5 |
| | Willi | - | 33.9 | 0.0 | 10.9 | 0.0 | 6.2 | 5.9 | 56.6 | - |

| **b)** | | **RECIPIENT - Close to the Wire Mesh Partitioning in TEST** | | | | | | | | |
|---|---|---|---|---|---|---|---|---|---|---|
| | | Juno | Signore | Soukie | Daisy | Paula | Peppi | Saul | Walter | Willi |
| **DONOR** | Caruso | 9.8 | 0.0 | 0.0 | 0.0 | 15.9 | 0.0 | 3.3 | - | - |
| | Daisy | - | 0.0 | 0.0 | - | 60.1 | 42.7 | 39.4 | - | - |
| | Paula | - | 14.1 | 2.3 | 0.0 | - | 0.0 | 0.0 | - | - |
| | Peppi | - | 0.0 | 0.0 | 0.0 | 6.5 | - | 0.0 | - | - |
| | Saul | - | 0.0 | 26.5 | 2.8 | 60.1 | 0.0 | - | - | - |
| | Walter | - | 17.1 | 0.0 | 0.0 | 2.5 | 46.5 | 19.5 | - | 18.2 |
| | Willi | - | 0.0 | 0.0 | 0.0 | 0.0 | 0.0 | 33.2 | 29.4 | - |

| **c)** | | **RECIPIENT - Close to the Exchange Table in TEST** | | | | | | | | |
|---|---|---|---|---|---|---|---|---|---|---|
| | | Juno | Signore | Soukie | Daisy | Paula | Peppi | Saul | Walter | Willi |
| **DONOR** | Caruso | 0.0 | 0.0 | 0.0 | 3.6 | 252.2 | 150.9 | 256.3 | - | - |
| | Daisy | - | 0.0 | 0.0 | - | 121.3 | 30.8 | 43.2 | - | - |
| | Paula | - | 267.8 | 56.4 | 0.0 | - | 344.1 | 513.6 | - | - |
| | Peppi | - | 0.0 | 0.0 | 129.9 | 113.7 | - | 291.4 | - | - |
| | Saul | - | 176.1 | 184.1 | 50.1 | 13.9 | 420.2 | - | - | - |
| | Walter | - | 0.0 | 0.0 | 0.0 | 14.0 | 0.0 | 40.2 | - | 244.7 |
| | Willi | - | 0.0 | 0.0 | 0.0 | 0.0 | 0.0 | 176.9 | 72.1 | - |

Figure S1 shows the azure-winged magpie recipient behavior scores separately for round 1 and round 2. Due to the small sample size (N=4), we were not able to run statistical analyses on only the magpies' data. Note however that in round 1 of each donor, the recipients spent more time close to the exchange table in the test than close to the wire mesh partitioning in both the test and the social control condition. This result was equivalent to the result obtained in the carrion crows. In round 2, in which we included additional training sessions, the results were the opposite: for each donor, the recipients spent less time close to the exchange table in the test than close to the wire mesh partitioning in both the test and the social control condition. It is possible that through the additional training sessions the recipients learned that they could not obtain any food at the exchange table without tokens and therefore spent less time in this location in round 2.

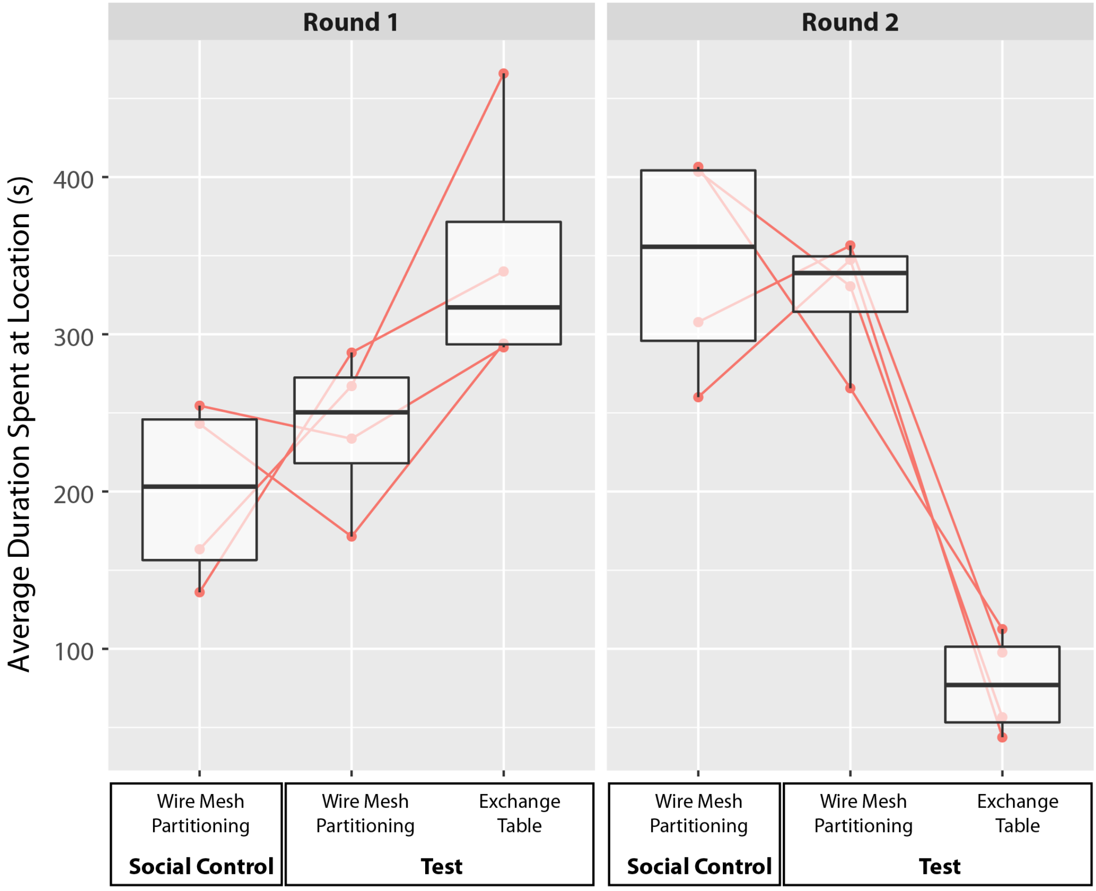


**Figure S1.** Duration that one or more azure-winged magpie recipient(s) spent at the given locations, averaged separately across the two sessions of round 1 and round 2 of each donor individual per condition. The box plots represent medians (horizontal lines), inter-quartile ranges (boxes), as well as minima and maxima (whiskers). All data are represented with dots.
